# Highlight report: Role of the ATP-releasing Panx channels in liver fibrosis

**Published:** 2019-01-22

**Authors:** Daniela Fernanda González Leiva

**Affiliations:** 1 IfADo - Leibniz Research Centre for Working Environment and Human Factors, Dortmund, GERMANY

## ⁯⁯

Recently Sara Crespo Yanguas and colleagues from the University of Brussel published a study about the role of pannexin1 in the pathogenesis of liver fibrosis (Crespo Yanguas et al., 2018[3]). Panx channels are known as mediators of ATP release (Dahl, 2015[4]). After injury cells may release ATP and uridine-5'-triphosphate into the extracellular space. The released ATP attracts immune cells to the area of damage (Davalos et al., 2005[5]; Chekeni et al., 2010[2]). In cardiac fibrosis cardiomyocytes have been shown to release ATP via pannexin1 which contributes to activation of fibroblasts (Dolmatova et al., 2012[6]). However, in liver the role of pannexin1 in liver fibrosis remains unknown. Therefore, the authors compared pannexin1 knockout and wild-type mice after CCl_4_ treatment for 8 weeks and after bile duct ligation (Crespo Yanguas et al., 2018[3]).

Interestingly, pannexin1 knockout mice showed reduced collagen content, stellate cell activation, and inflammation compared to wild-type mice. Therefore, the release of ATP seems to contribute to myofibroblast activation also in the liver. In contrast to the CCl_4_- fibrosis model, bile duct ligation led to more hepatocellular injury and a stronger immune response in the pannexin1 knockout than in wild-type mice.

It is not surprising that different consequences are observed in the CCl_4_ and the bile duct ligation models. CCl_4_ is a model of pericentral liver damage where a fraction of approximately 40 % of hepatocytes in the centre of the lobule are killed (Hoehme et al., 2010[16]; Hammad et al., 2017[14]; Bartl et al., 2015[1]). It seems plausible that the ATP released from these damaged hepatocytes activates stellate cells (Leist et al., 2017[18]). In contrast, bile duct ligation leads to a ductular response with proliferation of cholangiocytes, branching and looping of bile ducts, leading to a denser mesh of interlobular bile ducts around portal veins (Vartak et al., 2016[21]; Jansen et al., 2017[17]). Simultaneously, periportal fibrosis occurs (Ghallab et al., 2018[10]). It is interesting that this phenomenon is enhanced by the pannexin1 knockout, although the responsible mechanism still has to be elucidated.

Currently, hepatotoxicity *in vivo* (Stöber, 2016[20]; Du et al., 2017[7]; Reif et al., 2017[19]; Hammad et al., 2018[15]; Ghallab et al., 2016[9]) as well as mechanistic studies in hepatocyte *in vitro* systems represent very active research areas (Ghallab, 2017[8]; Godoy et al., 2013[11], 2015[12], 2016[13]). In this rapidly progressing field Sara Crespo Yanguas and colleagues made an important contribution by revealing the role of ATP-release channels in the pathogenesis of liver fibrosis.

## References

[1] Bartl M, Pfaff M, Ghallab A, Driesch D, Henkel SG, Hengstler JG (2015). Optimality in the zonation of ammonia detoxification in rodent liver. Arch Toxicol.

[2] Chekeni FB, Elliott MR, Sandilos JK, Walk SF, Kinchen JM, Lazarowski ER (2010). Pannexin 1 channels mediate 'find-me' signal release and membrane permeability during apoptosis. Nature.

[3] Crespo Yanguas S, da Silva TC, Pereira IVA, Maes M, Willebrords J, Shestopalov VI (2018). Genetic ablation of pannexin1 counteracts liver fibrosis in a chemical, but not in a surgical mouse model. Arch Toxicol.

[4] Dahl G (2015). ATP release through pannexon channels. Philos Trans R Soc Lond B Biol Sci.

[5] Davalos D, Grutzendler J, Yang G, Kim JV, Zuo Y, Jung S (2005). . ATP mediates rapid microglial response to local brain injury in vivo. Nat Neurosci.

[6] Dolmatova E, Spagnol G, Boassa D, Baum JR, Keith K, Ambrosi C (2012). Cardiomyocyte ATP release through pannexin 1 aids in early fibroblast activation. Am J Physiol Heart Circ Physiol.

[7] Du K, Farhood A, Jaeschke H (2017). Mitochondria-targeted antioxidant Mito-Tempo protects against acetaminophen hepatotoxicity. Arch Toxicol.

[8] Ghallab A (2017). Highlight report: Metabolomics in hepatotoxicity testing. EXCLI J.

[9] Ghallab A, Cellière G, Henkel SG, Driesch D, Hoehme S, Hofmann U (2016). Model-guided identification of a therapeutic strategy to reduce hyperammonemia in liver diseases. J Hepatol.

[10] Ghallab A, Hofmann U, Sezgin S, Vartak N, Hassan R, Zaza A (2018). Bile micro-infarcts in cholestasis are initiated by rupture of the apical hepatocyte membrane and cause shunting of bile to sinusoidal blood. Hepatology.

[11] Godoy P, Hewitt NJ, Albrecht U, Andersen ME, Ansari N, Bhattacharya S (2013). Recent advances in 2D and 3D in vitro systems using primary hepatocytes, alternative hepatocyte sources and non-parenchymal liver cells and their use in investigating mechanisms of hepatotoxicity, cell signaling and ADME. Arch Toxicol.

[12] Godoy P, Schmidt-Heck W, Natarajan K, Lucendo-Villarin B, Szkolnicka D, Asplund A (2015). Gene networks and transcription factor motifs defining the differentiation of stem cells into hepatocyte-like cells. J Hepatol.

[13] Godoy P, Widera A, Schmidt-Heck W, Campos G, Meyer C, Cadenas C (2016). Gene network activity in cultivated primary hepatocytes is highly similar to diseased mammalian liver tissue. Arch Toxicol.

[14] Hammad S, Braeuning A, Meyer C, Mohamed FEZA, Hengstler JG, Dooley S (2017). A frequent misinterpretation in current research on liver fibrosis: the vessel in the center of CCl4-induced pseudolobules is a portal vein. Arch Toxicol.

[15] Hammad S, Ellethy T, Othman A, Mahmoud HYAH (2018). Highlight report: hepatotoxicity prediction with Hep3B cells. Arch Toxicol.

[16] Hoehme S, Brulport M, Bauer A, Bedawy E, Schormann W, Hermes M (2010). Prediction and validation of cell alignment along microvessels as order principle to restore tissue architecture in liver regeneration. Proc Natl Acad Sci U S A.

[17] Jansen PL, Ghallab A, Vartak N, Reif R, Schaap FG, Hampe J (2017). The ascending pathophysiology of cholestatic liver disease. Hepatology.

[18] Leist M, Ghallab A, Graepel R, Marchan R, Hassan R, Bennekou SH (2017). Adverse outcome pathways: opportunities, limitations and open questions. Arch Toxicol.

[19] Reif R, Ghallab A, Beattie L, Günther G, Kuepfer L, Kaye PM (2017). In vivo imaging of systemic transport and elimination of xenobiotics and endogenous mol-ecules in mice. Arch Toxicol.

[20] Stöber R (2016). Pathophysiology of cholestatic liver disease and its relevance for in vitro tests of hepatotoxicity. EXCLI J.

[21] Vartak N, Damle-Vartak A, Richter B, Dirsch O, Dahmen U, Hammad S (2016). Cholestasis-induced adaptive remodeling of interlobular bile ducts. Hepatology.

